# The relationship between future self-continuity and intention to use Internet wealth management: The mediating role of tolerance of uncertainty and trait anxiety

**DOI:** 10.3389/fpsyg.2022.939508

**Published:** 2022-08-02

**Authors:** Rongzhao Wang, Xuanxuan Lin, Zetong Ye, Hua Gao, Jianrong Liu

**Affiliations:** School of Psychology, Fujian Normal University, Fuzhou, China

**Keywords:** Internet wealth management, future self-continuity, tolerance of uncertainty, trait anxiety, Internet finance

## Abstract

This study aimed to analyze the mediating effect of tolerance of uncertainty (TU) and trait anxiety (TA) on future self-continuity (FSC) and intention to use Internet wealth management (IUIWM) systems. A questionnaire survey was distributed online and a total of 388 participants completed questionnaire, The questionnaire included the following scales: Chinese version of the FSC, Intention to Use the Internet Wealth Management, TU, and TA. Pearson correlation was used to investigate the correlation coefficient between variables while the sequential regression method was used to analyze relationship between variables. To analyze the collected data, the SPSS 26.0 was used. A two-step procedure was applied to analyze the mediation effect. Confirmatory factor analysis (CFA) was conducted to test the measurement model. Afterward, the Maximum Likelihood method was used for path analysis, and the Bias-corrected Bootstrap method was used to investigate determine the estimated value and confidence interval of the mediating effect. To analyze the mediation effect, the Mplus 7.0 was used. The results showed that FSC positively predicted individuals’ Internet wealth management systems. Furthermore, TU and TA played complete serial multiple mediating roles between FSC and IUIWM. The role of TA and TU have negative impact on intention to use. This study provides a theoretical basis in personality psychology that Internet financial product suppliers can use to improve the attractiveness of their products. Product managers can subdivide users according to these personality traits to provide customized products.

## Introduction

Since 2013, when Yu’ E Bao ushered in an era of Internet wealth management in China, the country’s Internet Wealth Management market has developed rapidly (Zhao and Zhang, 2021). Internet wealth management is a system that the Internet as its medium for financial management. When the participation of financial intermediaries such as banks and securities brokers is considerably reduced, the supply and demand of funds can trade directly through information intermediary platforms to enable property gain for investors (Ren and Sun, 2017; Sun et al., 2020). Internet financial services, as an emerging financial investment and management technique, have attracted many individual users by offering savings products and money market funds with more robust returns. Average returns are higher than bank savings rates over the same period, and trading is more straightforward. These features make them an attractive alternative to bank savings (Wang and Tang, 2020). Benefitting from its advantages such as convenience, low threshold, and fragmented management, the number of Internet financial users has changed significantly. According to the data on the website of Tianhong Asset Management, which manages the Yu ‘E bao fund, Yu;’E bao now has 260 million users and held funds totaling about 620 billion RMB by the end of 2015 (Ren and Sun, 2017). Resultantly, the pace of change was so rapid that Internet wealth management profoundly changed the structure of finance with a subversive impact (Dong and Guo, 2015).

Although Internet finance has a short development time, it has exerted a great influence on China’s financial market though its characteristics and has gradually become a new form of business that cannot be ignored in the entire domestic financial ecosystem. It has also led to a remarkable change in the way and pattern of personal finance management.

Behavioral finance demonstrates that in addition to observed objective variables, psychological factors are important subjective variables affecting investors’ behavior choices (Kasemsap, 2015). Research on Internet financial management has focused more on qualitative analysis, such as the development model, *status quo* research and external influencing factor (Zhao and Zhang, 2021). As far as Internet financial products are concerned, there are few existing studies on what psychological factors will affect investors’ purchase of products and how. Moreover, for individuals who lack personal wealth management skills and are not driven to consider the future, the prevalence of consumerism, promotions, online sales, and so forth makes it easy to fall into a consumerism circle (Bartels and Urminsky, 2015); thus, investigating internal factors that affect individual financial management are urgently needed.

According to different service forms, Internet finance can be divided into three categories: Internet extension of traditional financial services (such as e-banking and online banking), financial Internet intermediary services (such as third-party payment and P2P online loan platforms), and Internet financial services (such as the Internet Fund and insurance sales platforms) (Ping and Chuanwei, 2012). In this study, we defined Internet wealth management as the purchase and use of Internet funds.

### Future self-continuity and Internet wealth management

Future self-continuity (FSC) reflects an individual’s present self and future self, influencing their future behavior (Ersner-Hershfield et al., 2009). It is an essential indicator of their access to long-term future development (Bartels and Urminsky, 2011) FSC is how an individual perceives a strong connection between the present self and the future self. The more robust the link, the stronger the individual’s FSC (Ersner-Hershfield et al., 2009; Liu et al., 2018) will be. Hershfield (2011) argued that FSC consists of three components: similarities to the future self, the vividness of the future self, and positive affect (positivity) toward the future self.

FSC has many effects on individual psychology and behavior. For example, individuals with high FSC more frequently choose to delay options in intertemporal decision-making tasks to obtain higher monetary benefits (Ersner-Hershfield et al., 2009). Furthermore, FSC significantly predicts anxiety and depression levels in individuals, with high FSC individuals showing lower levels of anxiety and depression (Sokol and Serper, 2019). In addition, individuals with high FSC are more rational in their consumption and show less impulsive consumption behavior (Bartels and Urminsky, 2015). FSC is also related to saving behavior. Individuals who perceive their future to be more similar to the present (high FSC) will exhibit less consumption behavior and increase investment in their savings. They perceive the future as a continuation of their present self and need to prepare for it (Ersner-Hershfield et al., 2009; Hershfield et al., 2011). Conversely, individuals who believe that their present self- differs from their future self- experience have a sense of separation between their present and future selves (Gelder et al., 2013). These individuals see no reason to prepare for their future selves and instead focus more on hedonistic consumption in the present moment (Pronin et al., 2008). If the present self is more considerate toward the long-term future self, the individual will set aside more resources to meet the future self’s needs. However, while previous studies of FSC and economic factors have focused on individuals’ saving behavior, little attention has been paid to individuals’ investment and financial behavior (Liu et al., 2018). With the development of the Internet economy, Internet wealth management has become popular (Zhao and Zhang, 2021). Therefore, we want to explore whether FSC will affect individuals’ Internet wealth management to some extent.

Internet wealth management reduces the participation of financial intermediaries because funds are traded directly through an information intermediary platform to achieve property gain for investors (Sun et al., 2020). Internet wealth management is popular among people with low-income and micro and small enterprises, which provide inclusive financial services to different classes of society, because of its low risk, low threshold, and high liquidity characteristics (Dong and Guo, 2015). The related savings products and money market funds have more robust average returns and lower overall risk than bank savings rates. Thus, they have become the leading choice of wealth preservation and appreciation for young and middle-aged people (Wang and Tang, 2020). Wang et al. (2020) argued that when individuals have a strong FSC, the difference between the anticipated state of the “future self” and the “present self” is not significant. Therefore, individuals with high FSC perception will prefer low-risk options and will not engage in high-risk investments to reduce the financial risk to their future selves. Therefore, we hypothesized that individuals with higher FSC are more likely to participate in Internet wealth management.

### Future self-continuity, tolerance of uncertainty and Internet wealth management

Tolerance of uncertainty (TU) refers to individual differences in emotional, cognitive, or behavioral reactions to uncertain situations (Buhr and Dugas, 2002; Zvolensky et al., 2010). Individuals faced with uncertain events worry because they cannot predict the consequences. Individuals who can manage uncertainty and respond positively are well adapted (Carleton, 2016). In contrast, individuals who cannot tolerate uncertainty and try to avoid it feel anxious and uneasy. TU reflects these inter-individual differences (Huang et al., 2014). TU is thought to be significantly related to generalized anxiety disorder (Counsell et al., 2017), social anxiety (Shannon et al., 2021), and memory bias (Francis et al., 2016). Adelman et al. (2016) showed that students with higher FSC were more thoughtful about future consequences. High FSC was significantly and positively associated with self-control (Qing et al., 2021). These individuals are better able to control anxiety when dealing with uncertainty (Tangney et al., 2004). Luhmann et al. (2011) found that individuals with low TU preferred the immediate option in an intertemporal choice task because waiting in an uncertain delayed option state was torturous.

Although most Internet wealth management benefits are more robust and the overall risk is low, small risks still exist (Dong and Guo, 2015). Since individuals with low TU have a low tolerance threshold for uncertain events, even when the probability of an uncertain event is small, they perceive it as threatening, intolerable, and anxiety-generating (Buhr and Dugas, 2002). This tendency may decrease their intention to use Internet wealth management (IUIWM) products. Individuals with high TU have a higher tolerance threshold for uncertain events, can accept the existence of certain risks, have a lower level of anxiety, and therefore may be more inclined to use Internet wealth management products. We take into account the mindsponge model, an emerging theory of transformative cultural values popular across multiple disciplines, which states that the individual mind acts like a sponge, absorbing new cultural intellectual values and eliminating outdated ideas in a multi-dimensional environment (Vuong and Napier, 2015). Through the mindsponge principle, we can understand the acceptance process of individuals for Internet financial management: First, individuals learn the basic information of Internet financial management, then evaluate it based on the existing knowledge and experience, and finally judge whether Internet financial management can help them better adapt to the uncertain future. Specifically, individuals with high FSC can overcome their fear of uncertainty, better build up their inclusion and trust in emerging things, and ultimately lead to a higher willingness to use Internet financial management. Therefore, we suggest that high TU individuals would have a greater tendency to use Internet wealth management products. FSC will affect the intention to use these products by influencing individuals’ TU.

### Future self-continuity, trait anxiety and internet wealth management

Trait anxiety (TA) is a relatively stable personality trait that refers to an individual’s propensity to be anxious when assessing external stimuli or dangerous situations differently (Sang et al., 2019). Individuals with high TA are more likely to exhibit anxiety and agitation when faced with uncertain situations (Endler and Kocovski, 2001). Lauriola and Levin (2001) noted that individuals with high TA tend to overestimate risks when considering their future. Even when the probability of adverse events is low, individuals with high TA show severe anxiety levels and have a significant attentional bias toward negative information (Loewenstein et al., 2001; Yu, 2020). Individuals with high TA are more conservative in making decisions and facing rewards, preferring to maintain the *status quo* rather than engage in tenuous wealth management investments (Pittig and Scherbaum, 2020). Even though the overall risk of Internet wealth management is low, for people with high TA, the very low probability of an adverse event is magnified. This risk makes them feel anxious. Thus, we assume that higher TA levels decrease individuals’ IUIWM products. Similarly, individuals with high FSC exhibit lower anxiety levels because heightened FSC awareness may help them to view life events from a broader perspective and realistically consider potential future self-experiences, thereby reducing an individual’s level of TA (Sokol and Serper, 2017, 2019).

Since the above research suggests that FSC can eliminate the negative effects of TA, TA will reduce the willingness to use Internet financial management. At the same time, FSC can effectively improve the rational consumption behavior of individuals, decision-making beneficial to the future self, and positive life expectations for the future (Bartels and Urminsky, 2011; Bartels and Urminsky, 2015; Rutchick et al., 2018). Therefore, can FSC eliminate the negative impact of TA on financial investment and direct it to a more positive direction? Thus, this study puts forward the hypothesis that although TA acts as a negative predictor, FSC can still predict Internet financial management through the intermediary path of TA.

### Future self-continuity, tolerance of uncertainty, trait anxiety and internet wealth management

According to risk-sensitivity theory, the primary goal of individuals in the face of risks is to avoid those options that will fail their goals, rather than necessarily pursue the maximization of benefits (Caraco et al., 1980; Mishra et al., 2017). Risk decision is a decision that is closely related to one’s current state and future goal. Since future goals are always associated with future self, risky decisions will depend on the extent to which there is a gap between my current state and my future goals. When it comes to financial decision risk, it refers to the change range of expected results caused by a financial decision. Why do some individuals avoid risk and others prefer it?

For example, option A’s benefits range from $1100 to $1300. The income range of option B is from $400 to $2,000, and the expected income of the two is the same ($1200 for both), but the risk of the latter is higher due to the larger range of change. If an individual’s goal is to eventually have $10,000, he is more likely to choose the low-risk option A if he already has $9,000. If the individual currently has only $8,000, he is more likely to choose the high-risk option B. If there is a much gap between the future self‘s goal and the present state, then the individual will prefer risk; Conversely, individuals will avoid risk if there is a small gap between their expected future self‘s goals and their present status. This is why we hypothesize that high FSC predicts more Internet financial management.

In decision-making, uncertainty is a key factor (Jaeger et al., 2013). We also take into account the vigilance-avoidance hypothesis (Mogg et al., 1997), Individuals who cannot tolerate uncertainty have certain cognitive biases, and will automatically regard vague or uncertain information as threatening and respond to it with certain negative repetitive thinking (Fergus et al., 2013). The attention to the threatening stimulus leads to the avoidance response, which hinders the objective evaluation of the stimulus and exacerbates the anxiety of the individual (Mogg et al., 2004). On the other hand, TU is often thought to be significantly associated with generalized anxiety disorder and negatively predicts its degree (Wright et al., 2016; Counsell et al., 2017; Heiden et al., 2019; Wilson et al., 2020). Song and Li (2019) found that TU was significantly correlated with TA and could predict individuals’ TA, with higher individual TU associated with lower TA. Low TU predicted higher TA levels (Mogg et al., 1997; Fergus et al., 2013). Ultimately, individual anxiety levels further enhance individual risk-averse behaviors (Mueller et al., 2010; Giorgetta et al., 2012; Charpentier et al., 2017).

Based on the above discussion, we believe that individuals’ FSC will affect individual’s financial risk preference. While TU and TA are affected by FSC, they also affect individuals’ financial risk preference. Specifically, (1) self-continuity means the stability of self in the time dimension, which ensures that the states and needs of the future self and the present self will not be greatly different. Therefore, combined with the risk sensitivity theory, individuals with high FSC prefer low-risk Internet financing. (2) Individuals with high FSC showed higher TU, while high TU predicted lower TA level, and eventually showed higher risk avoidance behavior and higher willingness to use Internet finance, a financial behavior with lower risk.

### The current study

In summary, since higher FSC is significantly and positively correlated with self-control, and individuals with higher self-control tend to have higher TU, FSC can influence individuals’ TU. Furthermore, as an essential factor affecting TA, it can predict individuals’ anxiety levels. In addition, anxiety levels influence individuals’ consumption and wealth management behaviors. Therefore, we proposed H1: individuals’ FSC positively predicts the IUIWM products; H2: Higher FSC is associated with higher TU and higher IUIWM products, with the TU having a mediation effect; H3: higher FSC is associated with lower TA and a greater tendency to use Internet wealth management products, with TA having a mediation effect; and H4: higher FSC is associated with higher TU for the future, lower TA, and a higher IUIWM products, with TU and TA having a serial multiple mediation effect.

This research has significant managerial and theoretical contributions. First, the relationship between FSC and IUIWM products has not been investigated. In addition, whether TU and TA act as mediating variables between FSC and intention has not been confirmed. Second, while FSC has been extensively studied in saving behaviors (Ersner-Hershfield et al., 2009), the role of FSC in shaping consumer behaviors has rarely been explored. Third, while external influence factors of individuals’ saving behaviors have received extensive attention from researchers, limited studies have investigated the role of personality characteristics and individual differences in saving behaviors.

## Materials and methods

### Sample and design

The methodological cross-sectional approach was adopted in this study. The participants were recruited through a convenient sampling technique. An online survey was conducted from September 2021 to November 2021 to collect date. Since most Internet finance users are aged 18–30 (Wang and Tang, 2020) and considering sampling convenience (She et al., 2021a), research sample screening criteria included (1) age 18–30 years old. (2) Knowledge of or have used Internet financial management. The prior sample size estimation was employed during the research planning state to avoid type I and type II errors (Beck, 2013; She et al., 2021b). The minimum sample size of 356 was required in this study based on 56 observed variables, a probability level less than 0.05, a power level of 0.8, and an effect size of 0.1 (Cohen, 2013). A total of 450 participants who met the criteria participated in the online self-report questionnaire through wjx.cn (a Chinese online survey platform). After removing 62 participants who failed to answer the screening questions correctly, 388 participants were included in the study. All participants were informed about participation and provided informed consent to participate. Table 1 presents participant demographic information.

**TABLE 1:** Participant demographics.

Demographics	Frequency	Percentage
Sex		
Men	120	30.92
Women	268	69.07
Age		
18–24	170	43.81
25–30	218	56.29
Education		
Primary school	3	0.77
High school	12	3.09
Undergraduate	354	91.24
Postgraduate	19	4.90

### Future self-continuity

We used the English version of the FSC scale compiled by Sokol and Serper (2019), which has 10 items. Each item is scored on a 5-point Likert scale, ranging from 1 to 5, with higher scores representing higher levels of FSC (e.g., “How similar are you now to what you will be like 10 years from now?”; “Do you like what you will be like 10 years from now?”). After the original FSC scale was translated into the Chinese version, we carried out a reverse translation to ensure that each item’s intention was accurately conveyed in the scale. Native English speakers were also invited to review the authenticity of the translated version. In addition, we invited 3 professors and 3 doctors of psychology to evaluate the content validity of the scale using a 4-point Likert scale, ranging from 1 to 4, with 1 being irrelevant and 4 being very relevant. The questionnaire had good content validity (*I-CVI* = 0.833–1, *S-CVI* = 0.9).

Based on FSC item analysis and exploratory factor analysis of 502 Chinese adults, the Chinese version of the FSC scale was revised, with 10 items reserved and summarized into three factors. CFA based on 380 Chinese adults showed that the three factors fit well (χ*^2^* = 44.011, *df* = 32; *CFI* = 0.991, *IFI* = 0.988, *RMSEA* = 0.027). The Chinese version of the FSC scale showed satisfactory Cronbach’s alpha (α = 0.823), retest reliability (*r* = 0.727), and convergent validity (*CR* = 0.90, *AVE* = 0.52). Scores of all items of FSC are averaged to give the total FSC score. Thus, scores on the total FSCQ range between 1 and 60, with higher scores indicating increased levels of FSC.

### Tolerance of uncertainty

Tolerance of uncertainty was assessed using the TU Scale (Huang et al., 2014). This scale includes three factors measured via 11 items, each rated on a 5-point Likert scale, ranging from “completely inconsistent” to “completely consistent,” with higher scores indicating lower TU levels (e.g., “I have to get rid of the uncertain state”; “I can’t relax if I don’t know what’s going to happen tomorrow”). In addition, we reverse-scored to facilitate analysis and research, with higher scores indicating higher TU levels. The scale authors reported a Cronbach’s α of 0.896, internal consistency α of 0.82, retest reliability of 0.78, and good structural validity (χ*^2^* = 67.15, *df* = 41; *NFI* = 0.96, *NNFI* = 0.98, *CFI* = 0.98, *RMSEA* = 0.043).

### Trait anxiety

The State- TA Inventory was used to measure TA (Shek, 1988) via 20 items of the TA subscale (e.g., “I feel calm”; “I feel secure”), including 10 reverse-scored items, each of which was rated on a 4-point scale, ranging from “little” to “almost always.” Higher scores indicate higher levels of TA. Cronbach’s α was 0.894. Previous studies have demonstrated that the questionnaire has good construct validity (Shek, 1993; Balsamo et al., 2013; Lin et al., 2020; Zhang et al., 2021).

### Intention to use internet wealth management

Intention to use Internet wealth management (IUIWM) was measured using the Internet Wealth Management Products Scale (Sun et al., 2020). This instrument assesses four factors using 14 items (e.g., “Internet wealth management products are credible”; “I am willing to understand and pay attention to the information related to Internet financial products”), each of which was scored on a 5-point Likert scale ranging from “completely disagree” to “completely agree.” Higher total scores indicate a higher IUIWM products. Cronbach’s α is 0.908, with good structural validity (χ*^2^*/*df* = 2.34; *GFI* = 0.94, *AGFI* = 0.91, *CFI* = 0.94, *NFI* = 0.89, *IFI* = 0.94, *RMSEA* = 0.067) (Sun et al., 2020).

### Data processing and analysis

Data were analyzed using IBM SPSS Statistics version 26 (IBM Corp, 2019). Statistical significance was set at *p* < 0.05. Means and standard deviations were used to describe the study variables. Associations between variables were analyzed using Pearson’s correlation coefficients. Stepwise regression was used to explore whether and how FSC, TU, and TA influenced the IUIWM. Then, a two-step procedure was applied to analyze the mediation effect (Anderson and Gerbing, 1988). Firstly, the measurement model, which involved four manifest variables, was tested to assess the goodness of fit represented by its explicit indicators. Secondly, if the index of measurement model met the requirements, the maximum likelihood estimation examined the structural equation modeling. We used Mplus Version 7 (Muthén and Muthén, 2017) to construct structural equation models with 5,000 bootstrap samples to identify the mediation effect further and estimate path coefficients (Baraff et al., 2016). Indirect effects were considered significant when the 95% bootstrap path coefficient confidence intervals did not cross zero. The path coefficients were accepted as significant at the 0.05 level.

## Results

### Descriptive statistics and correlations between variables

Table 2 shows the means, standard deviations, and correlations among variables. The correlation between TU and intent to use Internet wealth management (IUIWM) was not significant. However, all other variable pairs showed significant correlations.

**TABLE 2:** Descriptive statistics and correlations between variables (*n* = 388).

	*M* (*SD*)	1	2	3	4
1 FSC	36.42 (7.35)	1			
2 TU	20.65 (8.03)	0.25**	1		
3 TA	44.90 (9.13)	−0.48**	−0.55**	1	
4 IUIWM	50.63 (8.50)	0.18**	0.28	−0.26**	1

**p < 0.01.

### Regression analyses

First, IUIWM was taken as the dependent variable, and FSC, TU, and TA were taken as independent variables to conduct a stepwise regression analysis. FSC significantly predicted IUIWM (β = 0.184, *p* < 0.001). TU (β = –0.166, *p* < 0.01) and TA (β = −0.317, *p* < 0.001) had a significant negative impact on IUIWM. The results showed that FSC [*F*_(1, 386)_ = 13.479, *p* < 0.001, △*R*^2^ = 0.031] significantly positively predicted IUIWM. The results supported H1. The addition of TU did not significantly improve the variance of the regression model [*F(*_1, 385)_ = 6.792, *p* > 0.05, △*R*^2^ = 0.002], but adding TA resulted in a significant change in variance [*F*_(1, 384)_ = 12.883, *p* < 0.001, △*R*^2^ = 0.055]. This shows that TU and TA added to Model 3 had an impact on the predictive effect of FSC (Table 3).

**TABLE 3:** Stepwise regression analyses with intention to use Internet wealth management as the dependent variable.

	First step			Second step			Third step		
	*b*	*SE*	β	*b*	*SE*	β	*b*	*SE*	β
FSC	0.212	0.058	0.184***	0.218	0.06	0.188***	0.084	0.064	0.073
TU				–0.02	0.055	–0.019	–0.175	0.062	−0.166**
TA							–0.296	0.06	−0.317***
△*R*^2^	0.031***			0.002			0.055***		

**p < 0 .01, ***p < 0.001.

### Measurement model

Confirmatory factor analysis (CFA) was conducted to test the measurement model comprising the four manifest variables, the measurement model fit the observed data well: χ ^2^ = 7.344, *df* = 2, χ^2^/*df* = 3.67; *CFI* = 0.968, *TLI* = 0.904, *RMSEA* = 0.105, *SRMR* = 0.04.

### Structural analyses

**FIGURE 1:**
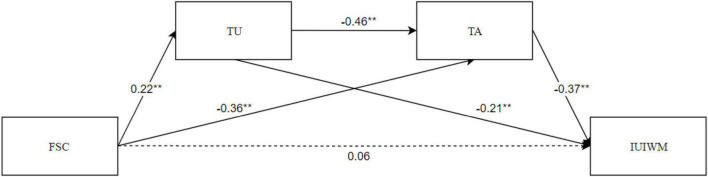
Serial multiple mediator model. **p < 0.01.

We used a structural equation model to test the mediating effect of TU and TA on FSC and IUIWM; the model fit index was in the acceptable range: χ ^2^ = 0, *df* = 0, χ^2^/*df* = 0; *CFI* = 1.000, *TLI* = 1.000, *RMSEA* = 0.000, *SRMR* = 0.000. The actual model is a saturated model, that is, all parameters to be estimated are exactly equal to the elements in the covariance matrix, and the degree of freedom is 0. Therefore, the fitting index is no longer estimated, and only the path coefficient is concerned (Kline, 2015). The results showed that FSC significantly positively predicted TU (β = 0.22, *p* < 0.01) and significantly negatively predicted TA (β = −0.36, *p* < 0.01. TU significantly negatively predicted TA (β = −0.46, *p* < 0.01) and IUIWM (β = −0.37, *p* < 0.01). The direct effect of FSC on IUIWM was not significant (β = 0.06, *p* > 0.05; Figure 1).

We used 5,000 bootstrapped samples to obtain the estimated bias-corrected confidence intervals of the three indirect effects. The indirect relationship between FSC and IUIWM through TU was significant. However, TU negatively predicted IUIWM, which is contrary to H2. The indirect relationship between FSC and IUIWM through TA was also significant. This significant mediating effect supports H3. Finally, the results showed that FSC was significantly related to IUIWM through serial multiple mediation of TU and TA, which supports H4 (Table 4).

**TABLE 4:** Bootstrapped indirect effects and 95% confidence intervals (CI) for the mediation model.

Model pathways	Estimated effect	SE	95% CI	
			Lower	Upper
FSC → TU → IUIWM	–0.048	0.0225	–0.103	–0.013
FSC → TA → IUIWM	0.134	0.0325	0.074	0.202
FSC → TU → TA → IUIWM	0.043	0.0143	0.020	0.077

In conclusion, the direct effect of FSC on IUIWM was not significant, indicating that TU and TA played a completely mediating role. Second, the positive effect of TA (59.65%) was greater than the negative effect of TU (21.39%). Finally, TU and TA played a sequential mediating role between FSC and IUIWM (18.95%).

## Discussion

The purpose of this study was to investigate the role of uncertainty tolerance (TU) and TA on FSC and IUIWM. Consistent with the hypothesis, FSC affects IUIWM through TU and TA. The mediating effect includes three paths: The independent mediating effect of TU, independent mediating effect of TA, serial mediating effect of TU and TA. The serial mediation results show that high FSC leads to high levels of TU, and led to low TA levels, and leads to higher IUIWM.

Internet wealth management is affected by various factors. The current results showed that FSC significantly and positively predicted IUIWM. When other mediating variables were not included, the influence of FSC on IUIWM was significant. This is consistent with previous research findings that FSC directly affects financial behavior (Wang et al., 2020). Individuals with high FSC are more rational consumers who show less impulsive consumption behavior. In addition, they prepare for their future by increasing their investment in savings (Bartels and Urminsky, 2015). Compared with traditional savings, Internet finance is a better choice for maintaining and increasing wealth, owing to its low risk, low threshold, and high liquidity (Dong and Guo, 2015). The findings from the current suggest that, the savings demand of individuals with high FSC is transformed into the demand for Internet finance, which ultimately improves their IUIWM. This also implies that individuals with high FSC are potential customers of Internet wealth management products. When product managers promote products, they should emphasize the attributes that products can provide reliable returns in the future to attract users.

On the other hand, the regression analysis showed that when TU and TA were added, the direct predictive effect of FSC on IUIWM became insignificant. TU mediated the impact of FSC on IUIWM, as individuals with high FSC had higher TU and lower IUIWM. This result is contrary to our hypothesis for TU, which proposed that TU would positively predict IUIWM. However, the regression analysis showed that TU had no significant ability to predict IUIWM under the condition of FSC control. The results of the mediating effect indicated that, after adjusting for FSC, TU negatively predicted IUIWM. Previous studies have found that individuals with high uncertainty tolerance will show more risk preferences and delayed option (Weber and Johnson, 2009; Luhmann et al., 2011). We found that individuals with high TU showed lower IUIWM. This may be because of the particularity of Internet wealth management, which is known to investors because of its small overall risk and relatively stable revenue (Dong and Guo, 2015). Individuals with high TU pay less attention to uncertain information in situations or events and are more active in attention, memory, and explanations of uncertain information (Francis et al., 2016). When faced with uncertain information, individuals with high TU tend to underestimate the probability of negative outcomes (Bredemeier and Berenbaum, 2008). When assessing investment and wealth management risks, individuals with high TU pay more attention to positive results, such as high returns, and ignore the possibility of high risks, which leads them to choose the traditional investment method with high returns and high risks. Therefore, a higher level of TU is an important factor leading to a lower level of IUIWM in individuals with higher FSC. This study is different from previous research results that emphasized the low-risk attributes of Internet wealth management products (Dong and Guo, 2015), suggesting that the return attributes of Internet wealth management products are also a key factor.

Further mediating effect tests also found that TA played a mediating role between FSC and IUIWM. Individuals with higher FSC had lower TA levels and higher IUIWM. Previous studies found that TA was low in individuals with high FSC (Sokol and Serper, 2017, 2019), which is consistent with the results of this study. We also found that IUIWM was higher in individuals with lower TA, which is consistent with the traditional view that high TA influences individuals to avoid risks and make conservative choices. Anxiety increases decision makers’ sensitivity to negative information processing, which leads to an attentional bias toward negative information, which further weakens their competitive motivation (Gambetti and Giusberti, 2012; Gu et al., 2015). When making investment and wealth management decisions, individuals with high TA consider adverse results such as losses more than they consider the probability of negative results and ultimately choose to reduce or stop investment behavior. TA not only directly predicts IUIWM but also mediates the effect of FSC on IUIWM. In this study, the mediating effect of TA between FSC and IUIWM was 59.65%, which is greater than that of TU by 21.39%. This suggests that more attention should be paid to the influence of TA on IUIWM.

We found that TU significantly predicted TA in a negative direction, which is consistent with previous studies (Fergus et al., 2013; Song and Li, 2019). Anxiety levels in individuals with low TU accumulate before eliminating uncertainty factors, which leads to higher levels of anxiety (Wright et al., 2016; Song and Li, 2019). This study found that TU and TA not only play separate mediating roles between FSC and IUIWM, but FSC also influences IUIWM through serial multiple mediation of TU and TA. With the addition of TU and TA, the direct effect of FSC on IUIWM was not significant. TU and TA played a complete mediating role, which shows that the influence of FSC on IUIWM is predominately through TU and TA. From the perspective of indirect effects, although TU and TA had a serial multiple mediating effect, they exhibited a suppressing effect. FSC reduced IUIWM by increasing TU (mediating effect size 21.39%). Conversely, TA was lower in individuals with high FSC, which resulted in a higher IUIWM (intermediary effect size 59.65%). The positive effect of TA was greater than that of TU. FSC decreased TA (serial multiple mediation effect size 18.95%) by increasing TU, resulting in an increase in IUIWM. According to the risk sensitivity theory, decision makers’ risk decisions are not necessarily to seek the results of utility maximization, but to avoid those results that cannot meet their own needs (Caraco et al., 1980; Mishra et al., 2017). In the scenario of financial risk decision-making, individuals with low FSC induced anxiety due to their low TU of investment uncertainty, thus improving their sensitivity to potential risks of Internet financial management, and producing attention bias and priority processing, and more inclined to negative interpretation of the results of processing. Therefore, in order to avoid loss or negative emotional experience and meet the need of their own safety, anxiety promotes individuals’ risk aversion, thus reducing their willingness to use Internet financial management.

### Theoretical implications

In terms of theoretical significance, this paper firstly enriches the research on financial risk decision-making, especially the influence on Internet online finance. Existing studies have mainly discussed the external influencing factors of Internet finance (Zhao and Zhang, 2021). This paper explores the internal influencing factors from an individual perspective. Secondly, this study constructed a series of mediation models, which can provide reference for related research. Future research can explore other internal influencing factors of Internet finance. Third, current study also demonstrates the applicability of FSC theory in China. At the same time, the revised Chinese version of the FSC Questionnaire can also be applied to other studies with Chinese subjects.

### Practical implications

In terms of actual impact, we provide practical operation plans for Internet financial products: Internet financial product managers need to improve product competitiveness and expand market scale through customer segmentation and improving customers’ experiences. Breakthroughs can be made in the following three aspects: First, FSC plays a particularly important role in promoting the intention to use, while the role of TA cannot be ignored. Product managers can subdivide users according to these two personality traits to provide customized products. Second, TU has a negative impact on intention to use. Individuals with high TU may prefer traditional financial products with high returns. Therefore, product managers can emphasize the profitability of Internet financial products rather than the low risk. Third, Vuong et al. (2022) found that the credibility of information influenced individuals’ evaluation of using online healthcare information. Since the credibility of information and uncertainty are opposite, individuals with low TU are more willing to choose products with more real information, therefore they are more likely to choose financial products with higher credibility, which also revealed that Internet wealth management products providers should pay more attention to the authenticity of their publicity to attract more investors. Last but not least, there will always be risks of policy failures and systemic risks once the implementation becomes widely accepted and widely adopted, but it does not mean that the research does not make sense (Vuong, 2018). Investment is risky and financial management should be cautious.

### Study limitations

This study has some limitations. First, only young people between the ages of 18 and 30 years participated in the study. Therefore, the study’s conclusions cannot be generalized to other age groups. This leaves scope for future research to study IUIWM in other age groups. Second, conclusions drawn from cross-sectional studies may apply only to a certain point in time; longitudinal follow-up studies are needed to confirm the results. Third, this study found that TU and TA played a complete mediating role in FSC and IUIWM. However, other factors may influence the relationship between FSC and IUIWM. Future research may identify other influencing factors.

## Conclusion

Although there is ample research on Internet wealth management, very few studies have focused on the role of personality psychology in people’s intent to use Internet wealth management. Our results supported the hypothesized mediation models regarding the relationship between FSC and IUIWM. First, FSC negatively influenced the IUIWM through the mediation of tolerance to uncertainty. Second, FSC, through the mediating role of TA, positively influenced the IUIWM. Third, TU and TA played complete serial multiple mediating roles between FSC and intention to use Internet wealth management. The positive effect of TA was greater than the negative effect of TU.

## Data availability statement

The raw data supporting the conclusions of this article will be made available by the authors, without undue reservation.

## Ethics statement

The studies involving human participants were reviewed and approved by the Ethics Committee of the School of Psychology, Fujian Normal University. The patients/participants provided their written informed consent to participate in this study.

## Author contributions

RW: project development, data collection, data analysis, and manuscript writing. XL: project development, data analysis, and manuscript editing. ZY: data curation, investigation, and resources. HG: manuscript editing and resources. JL: methodology, manuscript editing, and project administration. All authors contributed to the article and approved the submitted version.
